# Supplementary material for: Steady-state water exchange in neural tissue is primarily passive and through the phospholipid bilayer
Source: bioRxiv. 2026 Jul 21:2024.12.12.628254. Preprint. [Version 3] doi: 10.1101/2024.12.12.628254 (PMC13419834; doi:10.1101/2024.12.12.628254)
Supplement: Supplementary file 1 [file NIHPP2024.12.12.628254v3-supplement-1.pdf]

bioRxiv preprint doi: https://doi.org/10.1101/2024.12.12.628254; this version posted July 21, 2026. The copyright holder for this preprint (which was not certified by peer review) is the author/funder. This article is a US Government work. It is not subject to copyright under 17 USC 105 and is also made available for use under a CC0 license.

Supporting Information for

Steady-state water exchange in neural tissue is primarily passive and through the phospholipid bilayer

Nathan H. Williamson, Rea Ravin, Teddy X. Cai, Julian A. Rey, Peter J. Basser

Peter J. Basser.
E-mail: basserp mail.nih.gov

This PDF file includes:

Supporting text
Figs. S1 to S6

bioRxiv preprint doi: https://doi.org/10.1101/2024.12.12.628254; this version posted July 21, 2026. The copyright holder for this preprint (which was not certified by peer review) is the author/funder. This article is a US Government work. It is not subject to copyright under 17 USC 105 and is also made available for use under a CC0 license.

Supporting Information Text

1. ADC provides a proxy for diffusion and DEXSY-derived volume fractions

Simultaneous realtime measurements were used to test for correlations between the metrics ADC_y, AXR, f (restricted water fraction), c, and f_{ng} (non-Gaussian signal fraction). ADC_y, f, and c arise from static gradient spin echo (SGSE) diffusion experiments. ADC_y measures the average mobility of all water molecules in the sample on the timescales of diffusion encoding (here between 0.20 and 0.51 ms). f and c are measured from fitting $I/I_0 = f\exp(-\tau c)$ to the SGSE diffusion signal attenuation at $b \geq 60$ ms/μm^2 and are associated with localization or motional averaging of water near surfaces on the length scale of the gradient dephasing length ℓ_g (see Williamson, et al., Magn. Reson. Lett., 2023) Here, with the extremely strong $g = 15.3$ T/m static gradient produced by the low-field single-sided magnet, $\ell_g = 0.8$ μm (at 25°C). c is the rate constant for the decay of this localized or motionally-averaged component as a function of echo time. c is related to a restriction length scale in the case of motional averaging, or D_0 in the case of localization, and also affected by exchange. ADC is a standard measurement used clinically (although with longer diffusion timescales between 20 and 100 ms), whereas f and c require gradient strengths not currently accessible with the pulsed gradients of clinical systems. f_{ng} is derived from the DEXSY data (see Eq. 19 in Cai, et al., J. Magn. Reson., 2024). Correlations between ADC and f have not been studied before, although they are often inferred in two-component models for ADC. Such models may include a hindered component and a restricted component with apparent diffusion coefficients D_1 and D_2 respectively, leading to $\mathrm{ADC} = (1-f)D_1 + fD_2$. Hindrances reduce D_1 below the diffusion coefficient of freely diffusing water, $D_0 = 2.15$ μm^2/ms at 25°C. Water that is restricted on length scales similar to or smaller than l_g will have $D_2 \ll D_1$, leading to $\mathrm{ADC} = (1-f)D_1$ in the limit that $D_2 = 0$. Note that the slope and intercept here are equal to D_1, the diffusion coefficient of the more mobile compartment. We use correlations to test this model and understand what the metrics are measuring in this system.

We find strong linear correlations between ADC_y and f (Figs. S2D and Figs. S5D). The correlation is the same regardless of the type of perturbation (with/without ouabain and osmolytes). Slopes and y-intercepts are similar to D_0 consistent with the model. These results are consistent with the picture that f is associated with effectively immobile water and ADC_y is a proxy for f. This suggests that both ADC_y and f report cellular swelling and shrinking.

ADC_y and f_{ng} are strongly correlated under normal conditions, with slope and intercept slightly above D_0, but not correlated after ouabain treatment (Figs. S2F). During osmolyte perturbations, the correlation appears strongest when $\Delta\mathrm{ADC}_y$ and Δf_{ng} are compared (Figs. S5F). The slopes are very close to 1, as predicted by the simple model. Deviations here are expected, since ADC_y and f are global measures averaged over the entire sample, whereas f_{ng} is a local measure averaged over exchanging compartments.

2. Discussion about differences observed during the wash

Osmotic perturbations with 100 mM sucrose and with variable concentrations up to 200 mM yielded consistent results overall (Fig. 4C,E). Differences were observed during the wash from hypertonic media to normal media on samples not treated with ouabain. AXR recovered to stable values slightly below baseline after washing out the +100 mOsm solution, but dropped to low levels, similar to ouabain-treated values, after washing out the +200 mOsm solution. (compare Fig. 3C to Fig. 4A). It appears that the samples maintained homeostasis during the wash in the first case but not in the second. This could be because of the larger osmotic change in combination with a longer experiment time. 200 mM is more than cell RVI mechanisms can physiologically balance given the limited NaCl and KCl in the aCSF and may have damaged the cells. Additionally, intracellular penetration of sucrose may have caused rebound swelling when washed to normal media. For reference, the wash occurred at roughly 120 minutes in the 100 mM case and at roughly 300 minutes in the 200 mM case.

3. Derivation of EEC-based model to predict tonicity

In this section we derive a model relating a rate constant k (such as AXR) to tonicity. The model is based on an application of transition state theory to the dynamics of mixtures. We first define the the enhancement of exchange with tonicity relative to a state of minimum (hypo)tonicity as $\theta = k_t/k_0$. Within transition state theory, $\theta = \exp(-\delta G_t/RT)$ where $\delta G_t = |G_t - G_0|$ is the change in Gibbs free energy associated with the difference in osmolarity of impermeable solutes in the extracellular space which increase the tonicity (hence the subscript t). Changes in Gibbs free energy can be decomposed into enthalpic and entropic contributions by its thermodynamic definition $\delta G_t = \delta E_{at} - T\delta S_t$. Then

$$\begin{aligned}\theta &= \exp\left(\frac{-(\delta E_{at} - T\delta S_t)}{RT}\right) \\ &= \exp\left(\frac{\delta S_t}{R} - \frac{-\delta E_{at}}{RT}\right) \\ &= \exp\left(\frac{\delta S_t}{R}\right)\exp\left(\frac{-\delta E_{at}}{RT}\right).\end{aligned} \qquad [1]$$

To become the Arrhenius model, we explicitly define the connections between the change in entropy and the preexponential factors $\exp(\delta S_t/R) = A_t/A_0$, and between the change in enthalpy and the activation energies $\delta E_{at} = E_{at} - E_{a0}$. Here, A_t and

bioRxiv preprint doi: https://doi.org/10.1101/2024.12.12.628254; this version posted July 21, 2026. The copyright holder for this preprint (which was not certified by peer review) is the author/funder. This article is a US Government work. It is not subject to copyright under 17 USC 105 and is also made available for use under a CC0 license.

E_{at} are tonicity-dependent and A_0 and E_{a0} are evaluated when the system is at a a state of minimum (hypo)tonicity, defining $c = 0$. Then

$$\begin{aligned}\theta &= \frac{A_t}{A_0} \exp\left(\frac{-(E_{at} - E_{A0})}{RT}\right) \\ &= \frac{A_t}{A_0} \frac{\exp(-E_{at}/RT)}{\exp(-E_{a0}/RT)}\end{aligned} \quad [2]$$

Now the effect of tonicity on exchange can be considered in terms of its separate effect on δE_{at} and δS_t.

The analytical functions for δE_{at} and δS_t with respect to the osmotic concentration can be obtained by Taylor expansions,

$$\begin{aligned}E_{at} &= E_{a0} + \left.\frac{dE_{at}}{dc}\right|_{\Delta c \to 0} \Delta c + O(\Delta c^2) + ... \\ S_t &= S_0 + \left.\frac{dS_t}{dc}\right|_{\Delta c \to 0} \Delta c + O(\Delta c^2) + ...\end{aligned} \quad [3]$$

where $O(\Delta c^2) + ...$ refer to second- and higher-order terms and $|_{\Delta c \to 0}$ refers to the term being evaluated in the limit of $\Delta c \to 0$, i.e., $c_t \to c_0$. We limit ourselves to a first-order approximation so that a two-parameter model can be obtained, but with the downside that the model loses validity as Δc increases above dilute concentrations. We also drop the evaluated symbol for brevity (although still implied). Then

$$\begin{aligned}\delta E_{at} &\approx dE_{at}/dc \; \Delta c \\ \delta S_t &\approx dS_t/dc \; \Delta c.\end{aligned} \quad [4]$$

These two parameters can be calculated from measurements of E_a and A at $c = c_0$ and c_1 with $\Delta c = c_1$ by using the relations $dE_{at}/dc = (E_{a(c=c_1)} - E_{a(c=c_0)})/\Delta c$ and $dS_t/dc = \ln(A_{c=c_1}/A_{c=c_0})R/\Delta c$. These are related to virial coefficients for the entropy and enthalpy of activation by $[E_{at}] = (dE_{at}/dc)/R$ and $[S_t] = (dS_t/dc)/R$. These definitions are combined with Eq. 1 to obtain the final relationship

$$\theta = \exp(-\alpha \Delta c) \quad [5]$$

where

$$\begin{aligned}\alpha &= (\delta E_{at}/\Delta c RT) - (\delta S_t/\Delta c R) \\ &\approx (dE_{at}/dc)/RT - (dS_t/dc)/R \\ &\approx [E_{at}]/T - [S_t].\end{aligned} \quad [6]$$

From this model, it is also possible to calculate the tonicity Δc as

$$\Delta c = \ln(\theta)/\alpha. \quad [7]$$

For our system, by comparing E_a and A of samples after treatment with ouabain to samples after treatment with ouabain and 100 mM sucrose ($c = c_0$ and $c = c_1$, respectively), we find $[E_{at}] = 16.1$ K/mM and $[S_t] = 0.0686$ 1/mM, leading to $\alpha \approx -0.0146$ 1/mM at 25°C. Based on k_t under normal and k_0 from ouabain-treated conditions $\theta = 130/35$, and Eq. 7 predicts $\Delta c = 90$ mOsm.

4. Additional PLM and 3XM details

The pump–leak model (PLM) uses the finite difference method to approximate the fluxes of ions based on their governing differential equations for mass conservation. Na^+ and K^+ transport actively with a 3:2 stoichiometry. Na^+, K^+, and Cl^- transport passively based on their electrochemical potential. The net charge of the intracellular impermeants is $z = -1$. Water permeability is assumed to be much higher than ion permeability and is not modeled directly. Instead, the cell changes volume so that the intracellular osmolarity matches the extracellular osmolarity at the end of each timestep. Voltage is modeled based on the net charge of the intracellular ions and the (constant) capacitance of the membrane. Unless stated otherwise, simulation parameters were the same as in Kay (2017). Volumes and voltages were taken as the values obtained at the final timestep. This time was sufficiently long for systems to reach steady-state (if there existed a stable steady-state), as determined by volume and voltage not changing when total time was increased by a factor of 10.

An analytical equation for the steady-state ECS fraction (f_o) was developed based on an osmotic balance which includes the contribution of an uncharged, trapped ECS osmolyte (x_o);

$$f_o = 1 - \frac{(1-z)x_i}{w_{tot}(\pi - 2Cl_o \exp(FV/RT)) + x_o/f_o} \quad [8]$$

where x_i is number of moles of intracellular impermeants (i.e., solutes which are trapped in the ECS or ICS that can not cross the plasma membrane or communicate with the bathing media), π is the concentration of small osmolytes in the ECS (i.e.,

bioRxiv preprint doi: https://doi.org/10.1101/2024.12.12.628254; this version posted July 21, 2026. The copyright holder for this preprint (which was not certified by peer review) is the author/funder. This article is a US Government work. It is not subject to copyright under 17 USC 105 and is also made available for use under a CC0 license.

solutes that can not cross the plasma membrane but can communicate with the bathing media), Cl_o is the concentration of extracellular chloride, F is the Faraday constant, R is the ideal gas constant, and T is the absolute temperature. π and Cl_o are assumed equal to that of the bathing media. The ECS and ICS volumes sum to a fixed total volume, w_{tot}. While this model does not explicitly account for non-osmotic (e.g. hydrostatic and mechanical) pressure gradients which could become large when cells swell and push against one-another or when cells shrink and push on intracellular organelles, the trapped ECS and ICS impermeant parameters act as placeholders to effectively model this buildup of pressure in cases of extreme swelling or shrinking. This equation does not model ion flux and instead assumes a value for the voltage (V). We used -48 mV for normal conditions and -10 mV for the conditions modeling Na^+/K^+–ATPase inhibition with ouabain. First, the PLM was used to find the steady-state cell volume predicted under normal conditions. This, along with $f_o = 0.3$ defined w_{tot}. Then x_o was arbitrarily set to 1/50th of the moles of intracellular impermeants x_i. Then Eq. 8 was used to predict ECS fraction f_o and osmolarities of intracellular and extracellular impermeants at varying osmolarities for the $V = -48$ mV and -10 mV conditions.

A three-site exchange model (3XM) associated with an ECS compartment (**a**) and two ICS compartments (**b** and **c**) was developed for predicting ADC and AXR. Eq. 8 was used to define the fraction of compartment **a** ($f_c = f_o$). For simplicity, the fractions of the two ICS compartments were set equal, $f_b = f_c = (1 - f_a)/2$. The ECS compartment and a more mobile ICS compartment were both given $\mathrm{ADC}_a = \mathrm{ADC}_b = 1\ \mu\mathrm{m}^2/\mathrm{ms}$. A less mobile ICS compartment was given $\mathrm{ADC}_c = 0.1\ \mathrm{ms}/\mu\mathrm{m}^2$. Varying ADC values of the compartments affected the overall ADC, the size of effects on ADC, and the correlation between ADC and AXR but did not affect the overall interpretations or the EEC relationship. ADC was modeled as the sum of the ADC values of each compartment multiplied by their volume fraction, $\mathrm{ADC} = f_a\mathrm{ADC}_a + f_b\mathrm{ADC}_b + f_c\mathrm{ADC}_c$. AXR was modeled by simulating data from the DEXSY experiment and then fitting the exchange model to the data. DEXSY signals were modeled using an operator formalism $\mathbf{S} = [1,1,1] * OD_2 * OE * OD_1 * \mathbf{S}_0$, ignoring relaxation, where OD_2, OE, and OD_1 are the operators for the second diffusion encoding, mixing time, and first diffusion encoding of the DEXSY pulse sequence, respectively. This involved multiplication of matrix exponentials which used the function expm() in MATLAB 2024a. The operators for the first and second diffusion encoding blocks were $OD_{1,2} = \mathrm{expm}(-b_{1,2}\mathbf{D})$ with $\mathbf{D} = [\mathrm{ADC}_a, \mathrm{ADC}_b, \mathrm{ADC}_c]'.*\mathrm{eye}(3)$, where eye(3) is a 3×3 identity matrix. Equilibrium magnetization was $\mathbf{S}_0 = [f_c, f_b, f_a]'$. The exchange operator was $OE = \mathrm{expm}(-t_m\mathbf{K})$ with exchange matrix

$$\mathbf{K} = \begin{bmatrix} (f_b + f_c)k_t & -f_a k_t & -f_a k_t \\ -f_b k_t & f_a k_t + f_c k_g & -f_b k_g \\ -f_c k_t & -f_c k_g & f_a k_t + f_b k_g \end{bmatrix} \qquad [9]$$

including transmembrane exchange (k_t) and geometric exchange (k_g) pathways. k_t and k_g values were determined by the Arrhenius model (Eq. 1) with $T = 298.15$ K or varied in the simulation, using $E_a = 52$ kJ/mol and $\ln(A) = 26.62\ \mathrm{s}^{-1}$ for k_t and $E_a = 22$ kJ/mol and $\ln(A) = 12.52\ \mathrm{s}^{-1}$ for k_g. These $\ln(A)$ values were defined for each E_a using Eq. 2 and the slope and intercept values presented in the caption of Fig. 7. Parameters (timings, gradient, b_1 and b_2 protocol, mixing times, etc.) were set based on values used experimentally. AXR values were estimated using the same DEXR model analysis method described in the "NMR hardware, experimental protocol, and analysis methods" section.

bioRxiv preprint doi: https://doi.org/10.1101/2024.12.12.628254; this version posted July 21, 2026. The copyright holder for this preprint (which was not certified by peer review) is the author/funder. This article is a US Government work. It is not subject to copyright under 17 USC 105 and is also made available for use under a CC0 license.

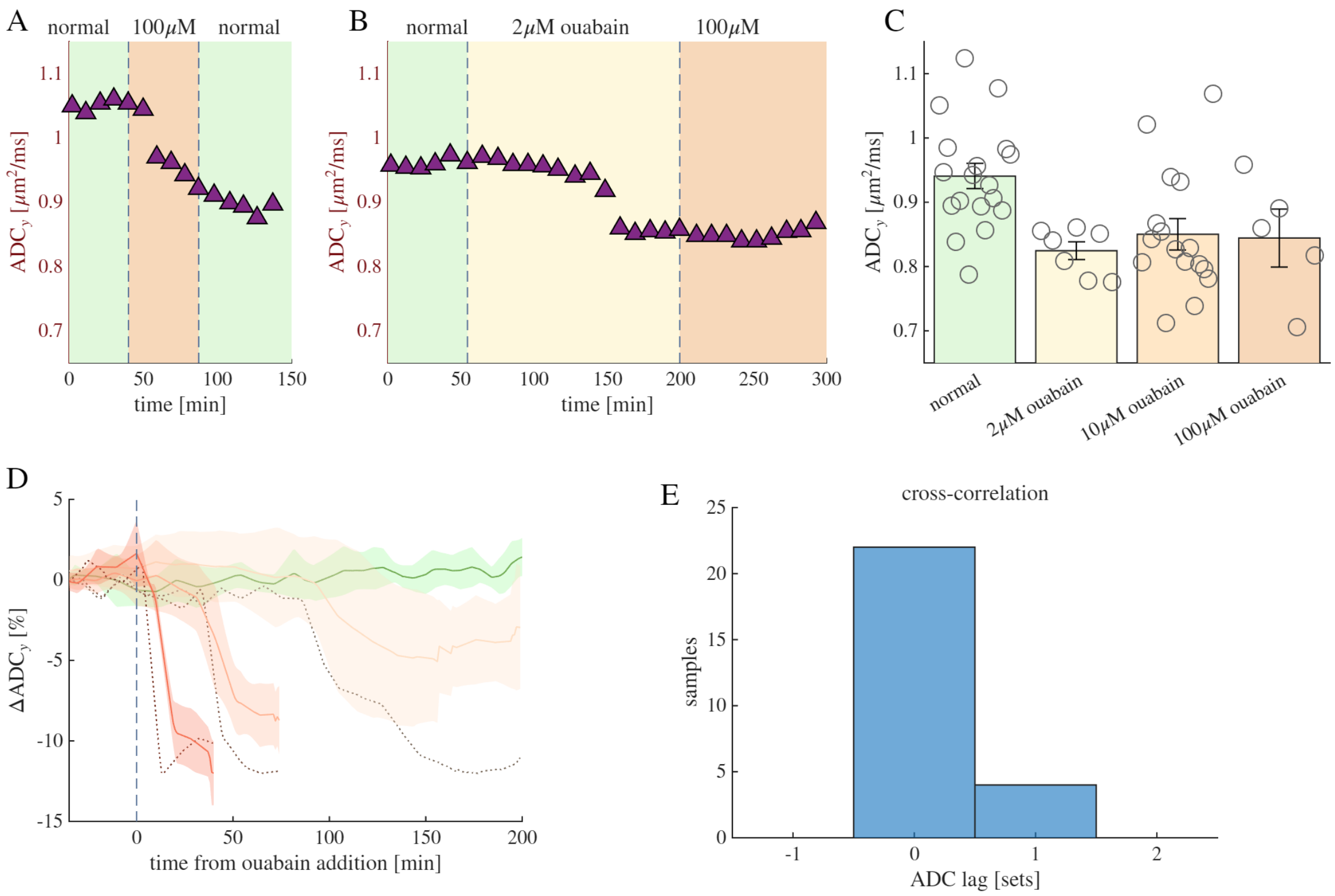

Fig. S1. ADC_y **and** AXR **are affected by ouabain simultaneously.** A–C) data reproduced from Fig. 1, but presenting the absolute values of ADC. Note the additional scatter between samples in (C) compared to when normalized by baseline values in Fig. 1C. D) Mean (solid lines) and standard deviation (shaded regions) of realtime ADC_y measurements from a control group under normal conditions (green) and groups treated with 2, 10, and 100 $\mu\mathrm{M}$ ouabain (lightest to darkest colors) plotted against the time after addition of ouabain. Dotted lines show the mean of AXR for each group, scaled to the maximum and minimum of the mean of ADC_y for comparison (refer to Fig. 1 for values) E) Histogram of the shift between the drop in ADC_y relative to the drop in AXR from a cross correlation analysis of 26 out of 28 samples for which there was a significant correlation between ADC_y and AXR ($cc > 0.2, p < 0.05$. Each set took 11 minutes to acquire and ADC_y measurements preceded AXR measurements. Analysis shows that ADC_y dropped either during the same set or the following set after AXR dropped, showing that ADC_y and AXR both dropped within 11 minutes of each other.

bioRxiv preprint doi: https://doi.org/10.1101/2024.12.12.628254; this version posted July 21, 2026. The copyright holder for this preprint (which was not certified by peer review) is the author/funder. This article is a US Government work. It is not subject to copyright under 17 USC 105 and is also made available for use under a CC0 license.

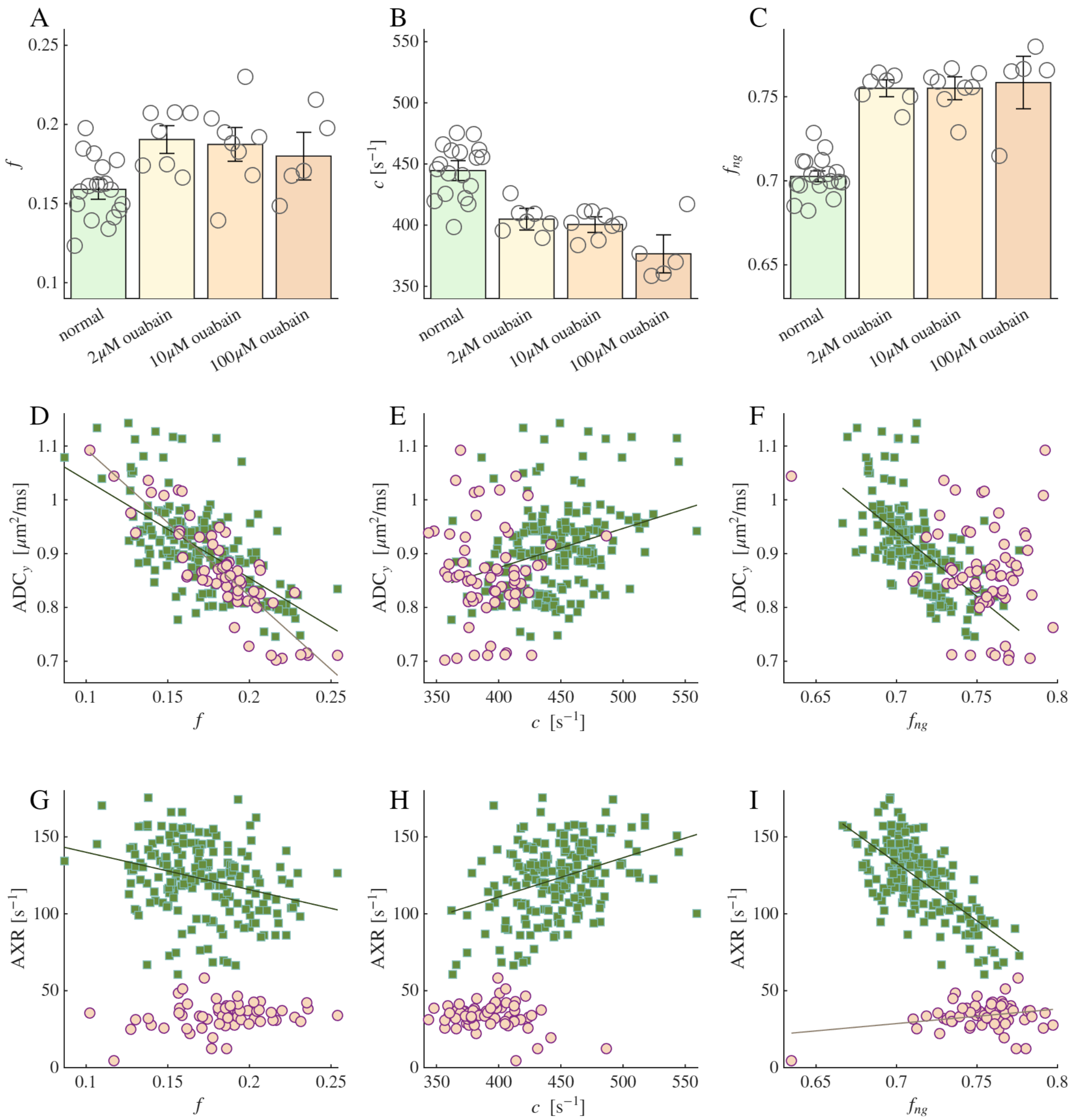

Fig. S2. Additional metrics and correlations from the effect of ouabain treatment A-C) Effect of ouabain on restricted fraction f and c from the intercept and slope of the SGSE diffusion signal at long times, and on the non-Gaussian signal fraction f_{ng} from the DEXSY data. D-I) Correlation of f, c, and f_{ng} vs. ADC_y and AXR. Data was grouped based on whether measurements occurred before (green squares) or after (pink circles) ouabain affected the exchange rate and dropped it below 60 s^{-1}. Linear fits are plotted when correlation is significant ($p < 0.05$). In (D), the data is significantly correlated for both groups ($p < 0.001$) with Pearson correlation coefficients (cc=) -0.64 before ouabain effect and cc=-0.87 after ouabain effect. Linear fits yielded $\mathrm{ADC}_y = -1.82f + 1.22\ \mu\mathrm{m}^2/\mathrm{ms}$ before ouabain effect and $\mathrm{ADC}_y = -2.72f + 1.36\mu\mathrm{m}^2/\mathrm{ms}$ after ouabain effect. In (F), the correlation is significant before the effect of ouabain, with ($p < 0.001$), cc=-0.65 and $\mathrm{ADC}_y = -2.4f_{ng} + 2.6\ \mu\mathrm{m}^2/\mathrm{ms}$, but is not significant after the effect.

bioRxiv preprint doi: https://doi.org/10.1101/2024.12.12.628254; this version posted July 21, 2026. The copyright holder for this preprint (which was not certified by peer review) is the author/funder. This article is a US Government work. It is not subject to copyright under 17 USC 105 and is also made available for use under a CC0 license.

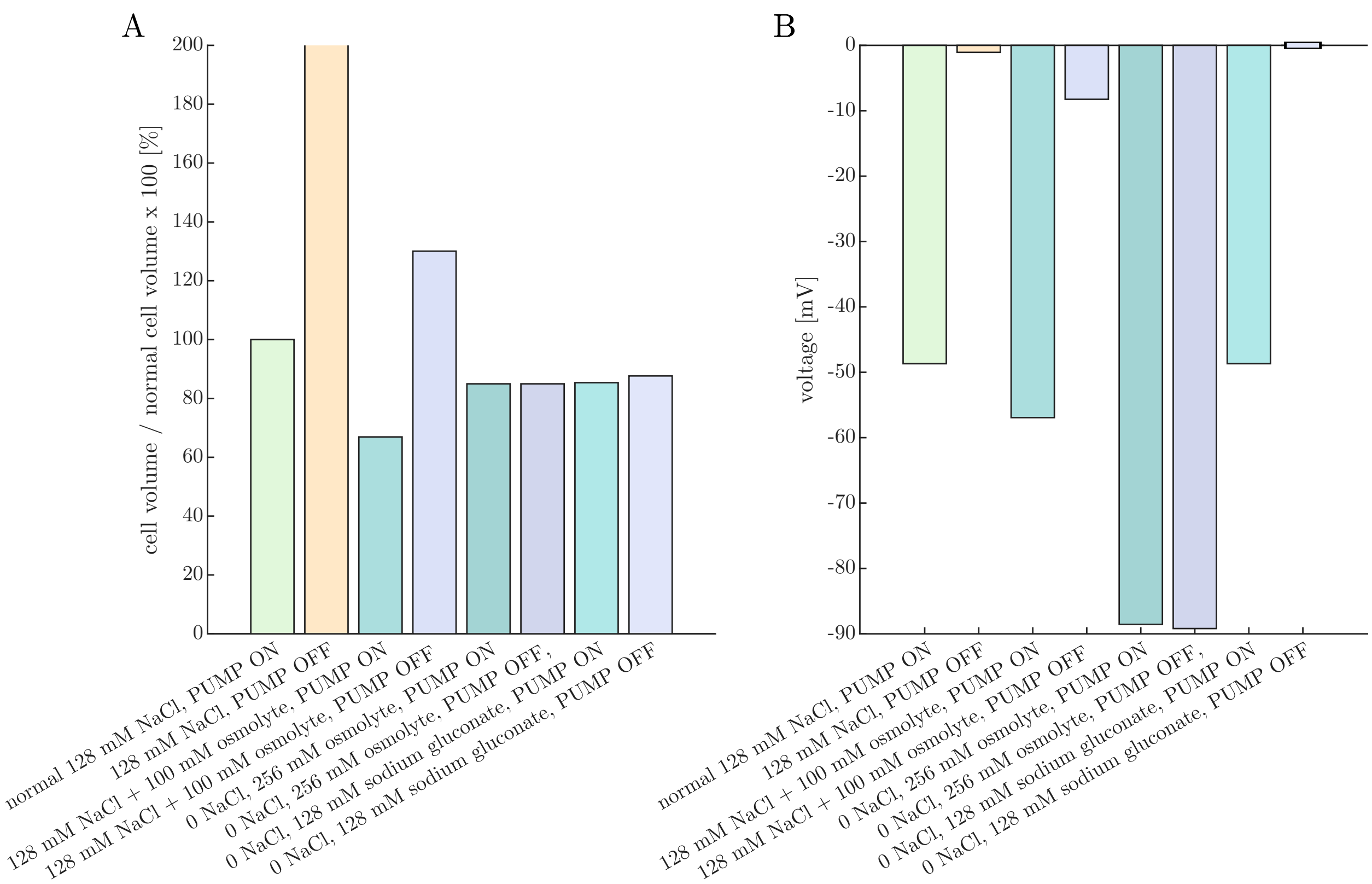

Fig. S3. Pump-leak model (PLM) provides predictions of cell volume and voltage under conditions studied experimentally. A) Percent change in cell volume and B) voltage predicted under various Na^+/K^+–ATPase PUMP ON or OFF and media conditions after the PLM has run to steady-state (if applicable). With the 128 mM NaCl (and 4 mM KCl) media and PUMP OFF condition, the cell continues to swell and does not reach a steady-state. Addition of an osmolyte (+100 mOsm) reduces the volume and hyperpolarizes the cell in the PUMP ON condition, and stabilizes the volume but does not recover the voltage with the PUMP OFF. The cell volume is reduced to a similar level in both the PUMP ON and PUMP OFF conditions when NaCl is replaced by either sucrose or sodium gluconate (modeled as uncharged and monovalent anionic impermeants in the bathing media, respectively). However, while voltage is similar between the PUMP ON and PUMP OFF conditions when NaCl is replaced by sucrose, full depolarization to $V = 0$ is predicted in the PUMP OFF condition with NaCl replaced by sodium gluconate.

bioRxiv preprint doi: https://doi.org/10.1101/2024.12.12.628254; this version posted July 21, 2026. The copyright holder for this preprint (which was not certified by peer review) is the author/funder. This article is a US Government work. It is not subject to copyright under 17 USC 105 and is also made available for use under a CC0 license.

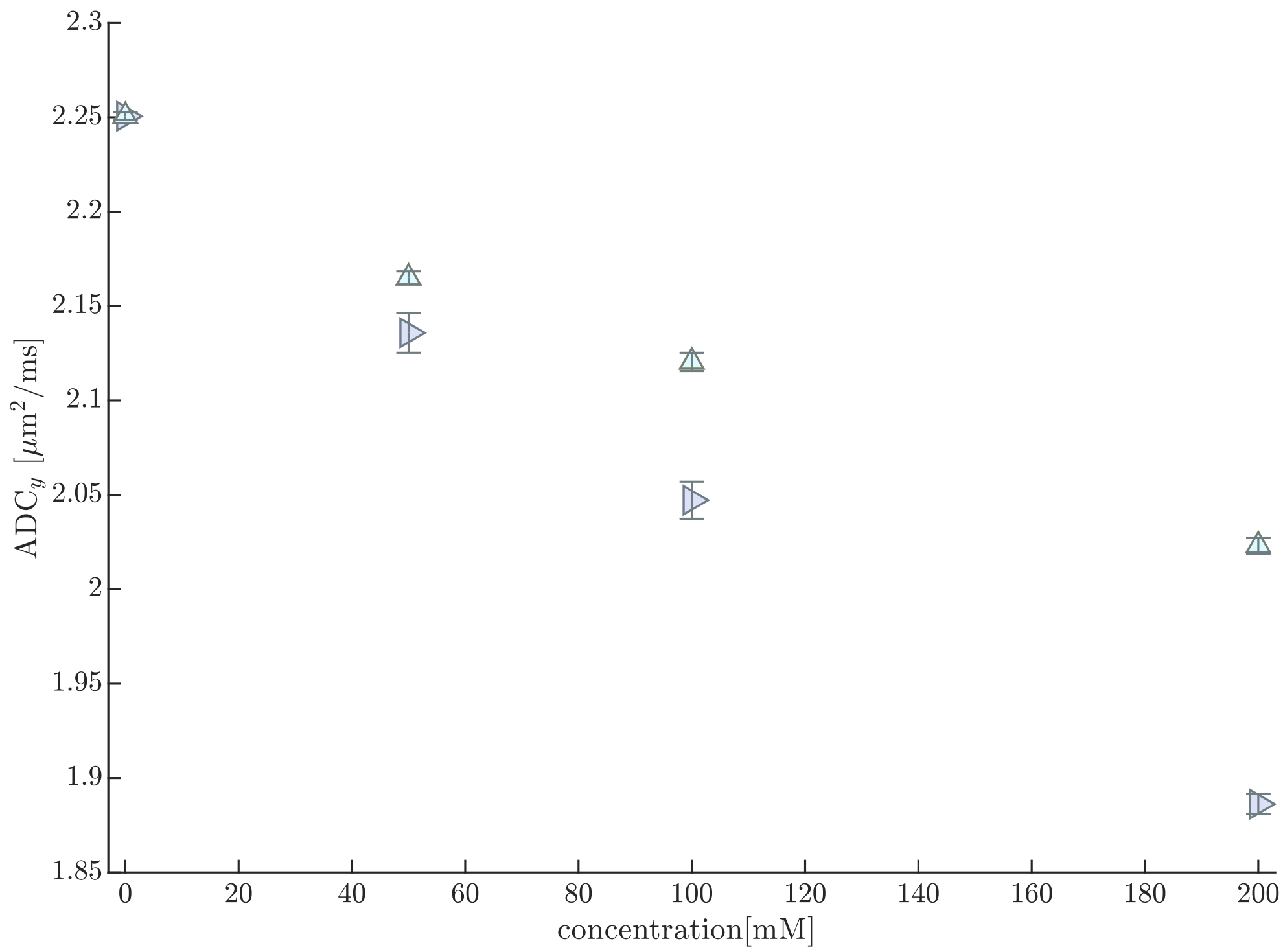

Fig. S4. Concentration-dependent effects of sucrose and mannitol on ADC of pure aCSF. Spin echo diffusion measurements were performed on aCSF at varying concentrations of sucrose (pink rightward-pointing triangles) or mannitol (aqua upward-pointing triangles) at 25°C without media circulation. At least three measurements were performed at each concentration using 8 τ values spaced linearly from $\tau = 105$ to $435\mu s$ ($b = 0.0129$ to $0.9159 \mathrm{ms}/\mu\mathrm{m}^2$), TR=2 s, and 2000 echoes. all other parameters were the same as standard diffusion measurements performed on spinal cords. Sucrose affects diffusion of water more than mannitol because it is a larger molecule (increased obstruction) and affects viscosity more (increased hydrodynamic interactions). This leads to the conclusion that the compounds' osmotic effects on tissue (cell shrinkage increasing ADC_y) are more significant than their viscous effects (reducing ECS diffusivity). Their viscous effects may become apparent at higher concentrations, e.g. at 200 mM, where the average ADC of samples treated with sucrose is less than those treated with mannitol, although not significantly.

bioRxiv preprint doi: https://doi.org/10.1101/2024.12.12.628254; this version posted July 21, 2026. The copyright holder for this preprint (which was not certified by peer review) is the author/funder. This article is a US Government work. It is not subject to copyright under 17 USC 105 and is also made available for use under a CC0 license.

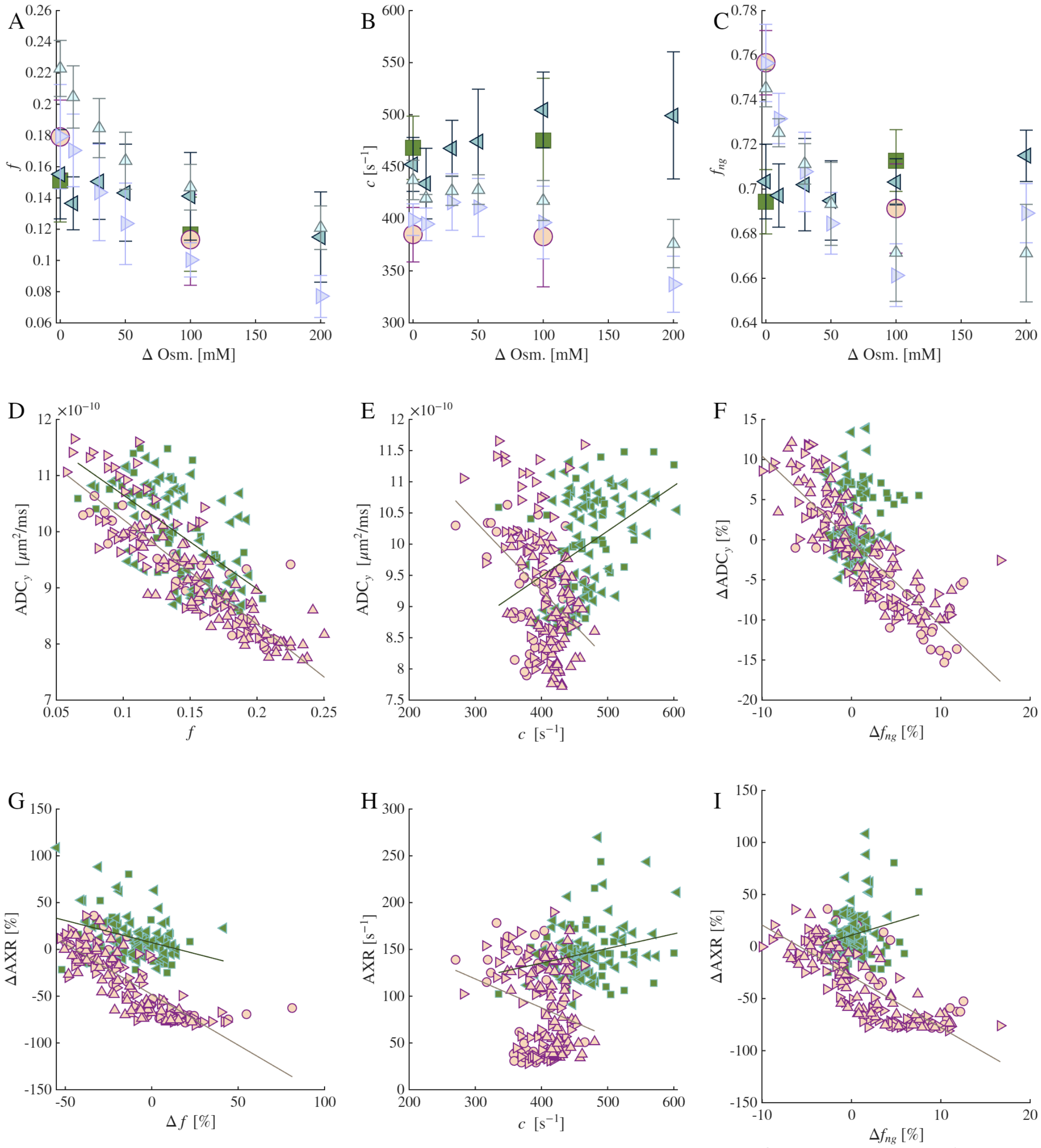

Fig. S5. Additional metrics and correlations from the effects of osmolytes without and with ouabain treatment A-C) Effect of osmolytes on f and c from the intercept and slope of the SGSE diffusion signal at long times, and on the non-Gaussian signal fraction f_{ng} from the DEXSY data. Refer to Fig. 1 for legend. D-I) Correlation of f, c, and f_{ng} vs. ADC_y and AXR. Data was normalized in cases where it tightened the correlation. In (D), cc= -0.61 without ouabain and cc=-0.87 with ouabain. Linear fits yielded $\mathrm{ADC}_y = -1.67f + 1.23\ \mu\mathrm{m}^2/\mathrm{ms}$ without ouabain and $\mathrm{ADC}_y = -1.87f + 1.21\mu\mathrm{m}^2/\mathrm{ms}$ with ouabain (note the slope slightly reduced from D_0). in (F), the correlation was insignificant without ouabain. With oabain, the correlation was significant ($p < 0.001$) with cc=-0.85 and linear fits yielded $\Delta\mathrm{ADC}_y = -1.05\Delta f_{ng} + 0.11\ \%$ (note the slope near 1). Note the sigmoidal correlations between Δf vs. $\Delta\mathrm{AXR}$ and Δf_{ng} vs. $\Delta\mathrm{AXR}$

bioRxiv preprint doi: https://doi.org/10.1101/2024.12.12.628254; this version posted July 21, 2026. The copyright holder for this preprint (which was not certified by peer review) is the author/funder. This article is a US Government work. It is not subject to copyright under 17 USC 105 and is also made available for use under a CC0 license.

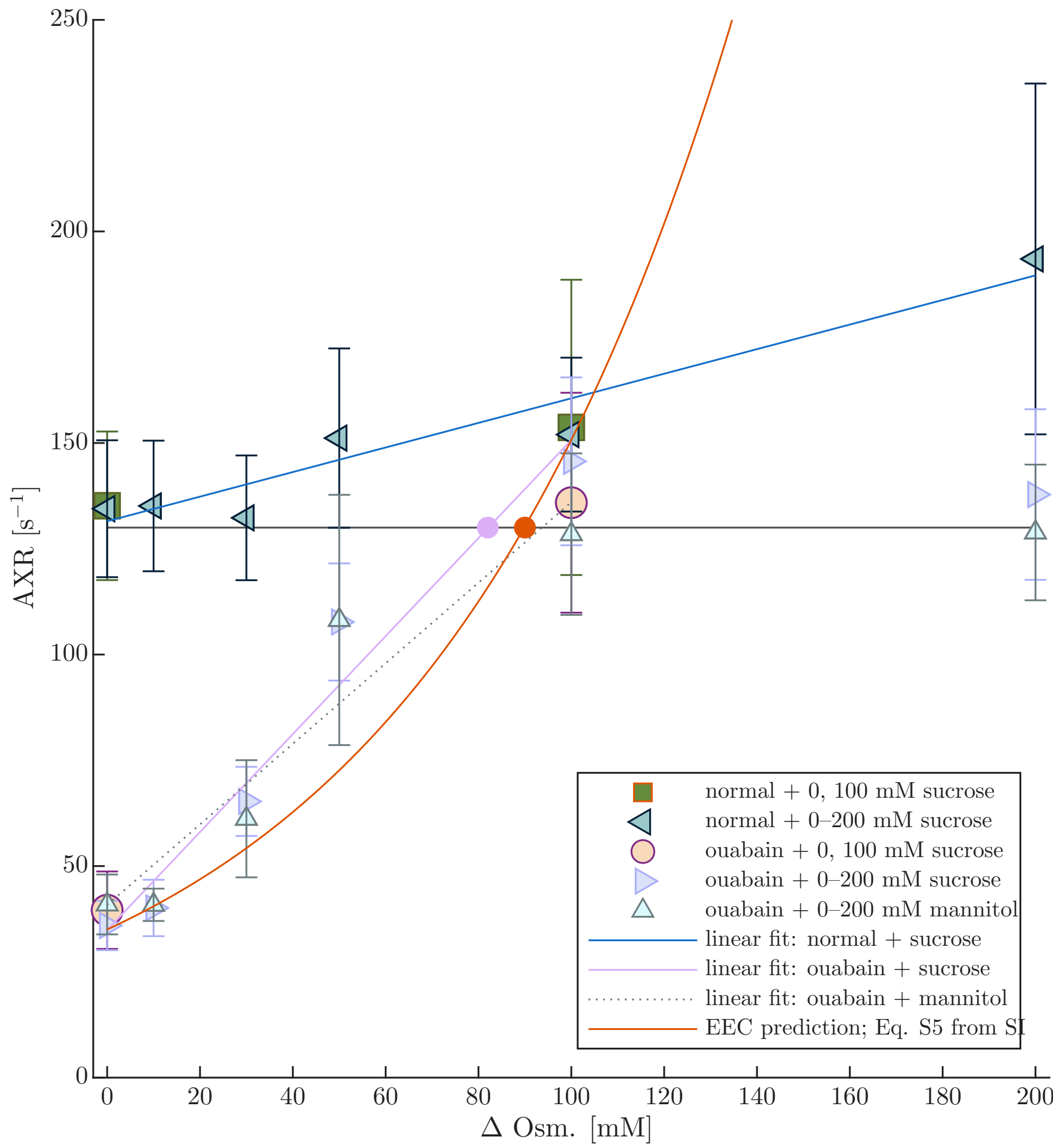

Fig. S6. Linear fits and EEC prediction for osmotic dependence of AXR Data reproduced from Fig. 4E from the main manuscript, with linear fits of the entire range (for normal + sucrose) or of the range from 0 to 100 mM for ouabain + sucrose and ouabain + mannitol, and the EEC prediction from Eq. 5 in SI section 3. The fit of the 0 to 100 mM for ouabain + sucrose data and the EEC prediction predict the AXR to equal the normal value (130 s^{-1}) at 82 and 90 mOsm., respectively, shown by the filled circles.
